# Characterization and functional evidence for Orf2 of *Streptomyces* sp. 139 as a novel dipeptidase E

**DOI:** 10.1007/s00253-024-13161-y

**Published:** 2024-05-08

**Authors:** Zhe Liu, Kemeng Li, Jialin Li, Zhuochen Zhuang, Lianhong Guo, Liping Bai

**Affiliations:** 1 CAMS Key Laboratory of Synthetic Biology for Drug Innovation, Institute of Medicinal Biotechnology, Chinese Academy of Medical Sciences & Peking Union Medical College, Beijing, 100050 China; 2 NHC Key Laboratory of Biotechnology of Antibiotics, Institute of Medicinal Biotechnology, Chinese Academy of Medical Sciences & Peking Union Medical College, Beijing, 100050 China

**Keywords:** Dipeptidase E, Orf2, *Streptomyces*, Exopolysaccharide, RNA-seq

## Abstract

**Abstract:**

Aspartyl dipeptidase (dipeptidase E) can hydrolyze Asp-X dipeptides (where X is any amino acid), and the enzyme plays a key role in the degradation of peptides as nutrient sources. Dipeptidase E remains uncharacterized in *Streptomyces*. Orf2 from *Streptomyces* sp. 139 is located in the exopolysaccharide biosynthesis gene cluster, which may be a novel dipeptidase E with “S134-H170-D198” catalytic triad by sequence and structure comparison. Herein, recombinant Orf2 was expressed in *E. coli* and characterized dipeptidase E activity using the Asp-*ρ*NA substrate. The optimal pH and temperature for Orf2 are 7.5 and 40 ℃; *V*max and *K*m of Orf2 are 0.0787 mM·min^−1^ and 1.709 mM, respectively. Orf2 exhibits significant degradation activities to Asp-Gly-Gly, Asp-Leu, Asp-His, and isoAsp-Leu and minimal activities to Asp-Pro and Asp-Ala. Orf2 contains a Ser-His-Asp catalytic triad characterized by point mutation. In addition, the Asp147 residue of Orf2 is also proven to be critical for the enzyme’s activity through molecular docking and point mutation. Transcriptome analysis reveals the upregulation of genes associated with ribosomes, amino acid biosynthesis, and aminoacyl-tRNA biosynthesis in the *orf2* mutant strain. Compared with the *orf2* mutant strain and WT, the yield of crude polysaccharide does not change significantly. However, crude polysaccharides from the *orf2* mutant strain exhibit a wider range of molecular weight distribution. The results indicate that the Orf2 links nutrient stress to secondary metabolism as a novel dipeptidase E.

**Key points:**

*• A novel dipeptidase E with a Ser-His-Asp catalytic triad was characterized from Streptomyces sp. 139.*

*• Orf2 was involved in peptide metabolism both in vitro and in vivo.*

*• Orf2 linked nutrient stress to mycelia formation and secondary metabolism in Streptomyces.*

**Supplementary Information:**

The online version contains supplementary material available at 10.1007/s00253-024-13161-y.

## Introduction

Actinomycetes are regarded as a valuable natural resource for clinical, agricultural, and biotechnological compounds, being major producers of secondary metabolites in the microbial world (Lacey and Rutledge 2022). *Streptomyces* belongs to Actinomycetes, which has been the source of approximately two-thirds of known antibiotics, as well as numerous microbial natural products with anticancer, antifungal, and immunosuppressive activities (Jose et al. 2021). In a previous study, a novel microbial extracellular polysaccharide Ebosin was isolated from *Streptomyces* sp. 139. Ebosin is an interleukin-1 receptor antagonist, capable of inhibiting acute and chronic proliferative inflammation and type IV delayed hypersensitivity reactions, as well as reducing the development of arthritis in rats and psoriasis in mice (Guo et al. 2021; Zhang et al. 2018). The structural analysis of Ebosin revealed its composition of rhamnose, arabinose, mannose, fucose, xylose, sialic acid, glucuronic acid, and glucose (Jing et al. 2003). The biosynthetic gene cluster for Ebosin had been identified previously (Wang et al. 2003).

**Fig. 1:**
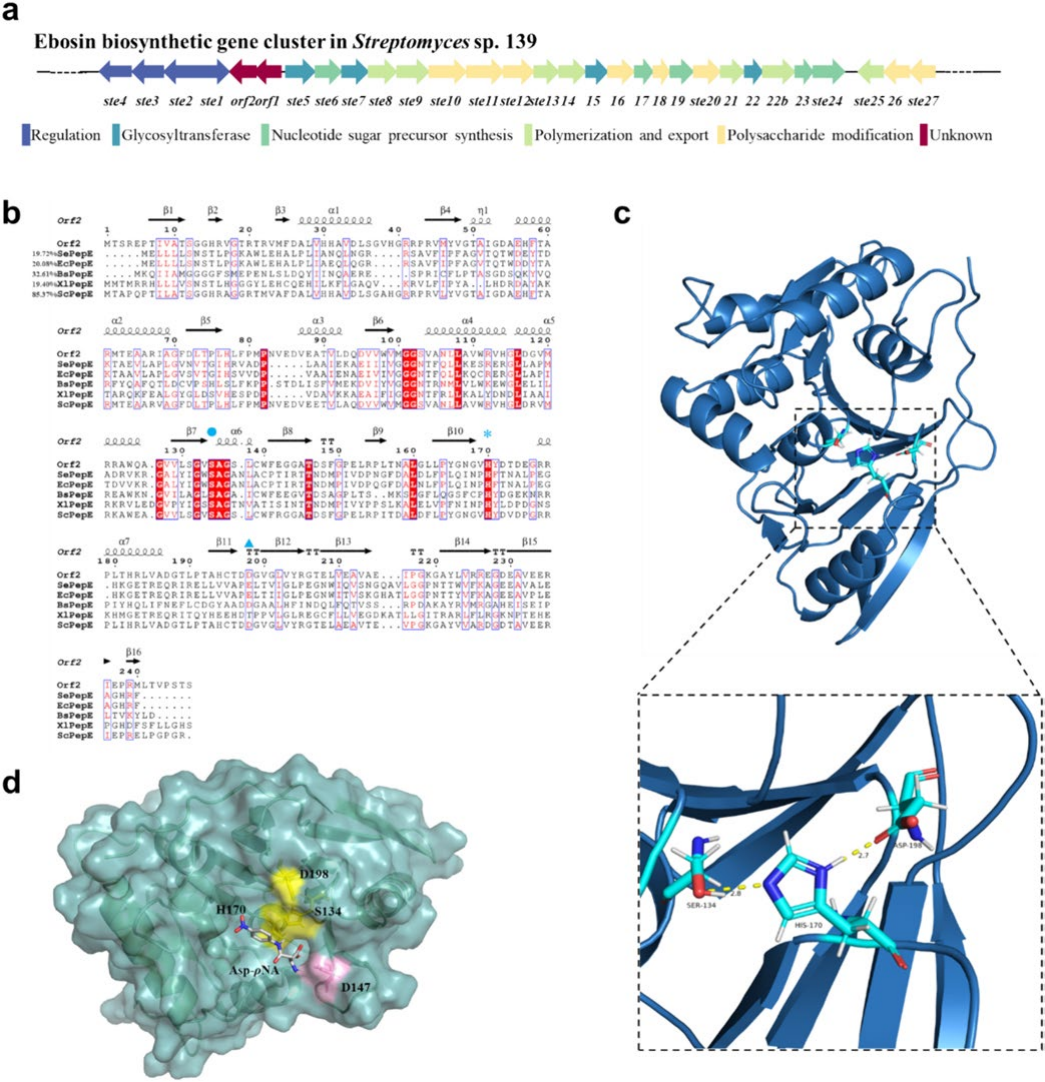
Structural and functional analysis of Orf2 protein. **a** Ebosin biosynthetic gene cluster in *Streptomyces* sp. 139. The function of each gene is as follows: regulation (*ste1*, *ste2*, *ste3*, *ste4*); glycosyltransferase (*ste5*, *ste7*, *ste15*, *ste22*); nucleotide sugar precursor synthesis (*ste6*, *ste17*, *ste19*, *ste23*, *ste24*); polymerization and export (*ste8*, *ste9*, *ste13*, *ste14*, *ste21*, *ste22b*, *ste25*); polysaccharide modification (*ste10*, *ste11*, *ste12*, *ste16*, *ste18*, *ste20*, *ste26*, *ste27*); unknown (*orf1*, *orf2*). **b** Multiple sequence alignment of the reported dipeptidase E family proteins. The Ser in the catalytic triad of dipeptidase E is marked by ●, and His is relatively conserved and marked by *. The last amino acid residue of the catalytic triad is typically Glu/Asp and is marked by ▲. Three catalytic residues in Orf2 were S134, H170, and D198, respectively. SePepE (19.72%) is derived from *Salmonella enterica* (UniProt ID: P36936). EcPepE (20.08%) is derived from *Escherichia coli* (UniProt ID: P0A7C6). BsPepE (32.61%) is derived from *Bacillus subtilis* (UniProt ID: P71089). XlPepE (19.40%) is derived from *Xenopus laevis* (UniProt ID: Q91642). ScPepE (85.37%) is derived from *Streptomyces coelicolor* (UniProt ID: Q9L121). Sequence percent identity to Orf2 is indicated in parentheses. **c** Protein structure and catalytic triad of Orf2. **d** Molecular docking of Orf2 and Asp-*ρ*NA.

Various *ste* genes had been functionally characterized by our lab: *ste1* and *ste2* for regulating exopolysaccharides production and phenotype of *S* sp. 139 (Bai et al. 2013; Geng et al. 2022); *ste5*, *ste7*, *ste15*, and *ste22* for bifunctional glycosyl-1-phosphate transferase, fucosyltransferase, glucosyltransferase, and rhamnosyltransferase, respectively (Chang et al. 2009; Li et al. 2010; Qi et al. 2015; Zhang et al. 2006); and *ste8* and *ste9* for polymerization and export of exopolysaccharides (Yang et al. 2013; Zhang et al. 2014). However, the function of the Orf2 protein encoded by the *orf2* gene, situated between *ste1* and *ste5* (Fig. 1a), remains unclear. Based on database searching, Orf2 belongs to the non-classical serine dipeptidase E protein family (PepE, EC 3.4.13.21, MEROPS ID: S51.001), members of which show specificity toward dipeptides with an N-terminal Asp (Asp-X, where X can be any amino acid) (Lassy and Miller 2000). Interestingly, dipeptidase E from *Streptomyces* has not been reported so far.

Dipeptidase E was first discovered in *Salmonella typhimurium* (Carter and Miller 1984); later, a homologous enzyme was found in *Synechocystis* sp. PCC6803, which can hydrolyze cyanophycin (multi-L-arginyl-poly-L-aspartic acid) into Asp-Arg (Richter et al. 1999). Subsequently, homologous dipeptidase E has been identified in other species, such as *Escherichia coli* (Conlin et al. 1994), *Listeria monocytogenes*, *Deinococcus radiodurans* (Yadav et al. 2019), *Xenopus laevis* (Kumar et al. 2022), and *Lactococcus lactis* (Kuerman et al. 2023). In this study, the characterization of Orf2 aims to elucidate its biological function in *S* sp. 139, providing valuable insights for the development of the dipeptidase E family.

## Materials and methods

### Strains, plasmids, primers, and growth conditions

The bacterial strains, plasmids, and primers (BGI Tech Solutions, Beijing, China) used in this study are listed in Table 1. *S* sp. 139 is isolated from a soil sample in China and kept in the China General Microbiology Culture Collection Center (strain number CGMCC 0405) with a fully sequenced genome (accession no. CP043959) capable of producing Ebosin. Both *S* sp. 139 wild-type strain and the *orf2* mutant strain were cultured in liquid medium tryptic soy broth (TSB, Solarbio Life Science, China) or TSB supplemented with 0.5% glycine and incubated at 28 °C with shaking (220 rpm). For solid medium cultures, the strains were cultured on 139 media (2.0% glucose, 0.05% asparagine, 0.05% K_2_HPO_4_, 2.0% agar) at 28 °C. *E. coli* strains were grown aerobically at 37 °C in Luria–Bertani (LB) medium or LB solidified with 1.5% agar, supplemented with appropriate antibiotics. The antibiotics used were 100 μg/mL kanamycin, 50 μg/mL apramycin, or 50 μg/mL thiostrepton, depending on the strain. For *E. coli* ET12567, an additional supplementation of 25 μg/mL kanamycin and 25 μg/mL chloramphenicol was used.

**Table 1:** Strains, plasmids, and primers used in this study

Strains or plasmids	Description	Source
Strains		
*Streptomyces* sp. 139	Wild type	Our lab
*Streptomyces* sp. 139 Δ*orf2*	*orf2* mutant of *Streptomyces* sp.139	This study
*Escherichia coli* DH5α	Used for plasmid screening and propagation, F^-^φ80d *lac*ZΔM15Δ(*lac*ZYA-*arg*F)U169 *end* A1 *rec*A1 *hsd*R17 (r_k_^-^, m_k_^+^) *sup*E44λ-*thi*-1 *gyr*A96 *rel*A1 *pho*A	TransGen Biotech
*Escherichia coli* BL21(DE3)	Used for protein expression, F^-^ *omp*T *hsd*S_B_(r_B_^-^m_B_^-^)*gal dcm*(DE3)	TransGen Biotech
*Escherichia coli* ET12567	Methylation-deficient *E. coli* dam− dcm− hsdM	(MacNeil et al. 1992)
Plasmids		
pET30a (+)	Carrying an N-terminal His•Tag®/thrombin/T7•Tag® configuration plus an optional C-terminal His•Tag sequence	Novagen
pET28a (+)	Carrying an N-terminal His•Tag®/thrombin/T7•Tag® configuration plus an optional C-terminal His•Tag sequence	Novagen
pET30a-*orf2*	pET30a (+) with *orf2*	This study
pET28a-*orf2*S134A	pET30a (+) with *orf2*S134A	This study
pET28a-*orf2*D147A	pET30a (+) with *orf2*D147A	This study
pET28a-*orf2*H170A	pET30a (+) with *orf2*H170A	This study
pET28a-*orf2*D198A	pET30a (+) with *orf2*D198A	This study
pKC1139	Shuttle plasmid (*E. coli*–*Streptomyces*), pSG5, pBR322, *aac*[3]IV *lac*Za *ori*TRK2, Apr^r^	(Bierman et al. 1992)
pKC1139-Δ*orf2*	pKC1139-derived plasmid carrying F1, *tsr* and F2 fragments, Apr^r^ Thio^r^	This study
T-*tsr*	T vector carrying thiostrepton resistance gene	Our lab
Primers		
*orf2*-F	CGGGATCCATGACGTCACGGGAACCCA	
*orf2*-R	CCGCTCGAGTCAGCTCGTCGAGGGGAC	
F1-F	CGACGGCCAGTGCCAGTCATGTGAGCGGTGGATGGACT	
F1-R	CATATGAAGTATTCGCCTTCTAGACGTCATGACCGTGATCGTAGGGG	
F2-F	TTCGTCAGTGATGATCATCTAGACTCACCGTCCCCTCGACGA	
F2-R	CAGCTATGACATGATTACGGTGAAACGCGGGCGGAGAA	
T1-F	GCATACGCGAAGGACCATCT	
T1-R	CTCCCGCTACAAGTTCACCG	
T2-F	ACAACGTGACCAACCTGTCG	
T2-R	CAGTCATGGTCGTCCTACCG	
T3-F	ATGAGCCAGACCGAGTCTCT	
T3-R	CAAATTGACGACACGCCGTT	

The underlined sequence is the restriction site used in constructing plasmids

### General DNA manipulation

The isolation of *E. coli* plasmid DNA and standard recombinant DNA techniques were performed as described previously (Green and Sambrook 2001). Likewise, *Streptomyces* genomic DNA was isolated following the method mentioned earlier (Kieser et al. 2000).

### Cloning, expression, and purification of Orf2 and mutant proteins in *E. coli*

The *orf2* gene was amplified using primers *orf2*-F/*orf2*-R (Table 1) and ligated into pET30a (+), yielding plasmids pET30a-*orf2*. Mutant Orf2 protein genes were synthesized by Tsingke Biotechnology (Beijing, China) and then ligated into pET28a (+), resulting in plasmids pET28a-*orf2*S134A, pET28a-*orf2*D147A, pET28a-*orf2*H170A, and pET28a-*orf2*D198A, respectively. These recombinant plasmids were transformed into *E. coli* BL21(DE3). After growing cultures of the recombinant strains to an optical density at 600 nm of 0.6~0.8, cells were induced with 1 mM IPTG for 3.5 h at 37 °C. Further incubation at 18 °C and 220 rpm for 18–20 h followed. Cells were collected by centrifugation (4000 × *g*, 20 min, 4 °C), resuspended with 10 mM imidazole and 500 mM NaCl in 50 mM Tris-HCl buffer (pH 7.5), and lysed using FastPrep-24^tm^5G (MP Biomedicals, USA). After centrifugation (12,000 × *g*, 30 min, 4 °C), the supernatant containing soluble proteins was manually purified using Ni-NTA Superflow columns (Qiagen, Germany) by gravity with gradient of imidazole (20, 40, 250, and 500 mM) and 500 mM NaCl in 50 mM Tris-HCl buffer (pH 7.5), followed by ultrafiltration into PBS buffer (8 mM Na_2_HPO_4_, 136 mM sodium chloride, 2 mM KH_2_PO_4_, 2.6 mM potassium chloride, pH 7.2–7.4).

Purified protein was analyzed by SDS-PAGE. Samples were heated at 100 °C for 10 min with 5 × SDS-PAGE loading buffer (Solarbio Life Science, China) and cooled down on ice. 10% One-step PAGE Gel Fast Preparation Kit (Meilunbio, China) was used to prepare the gel, perform electrophoresis at a constant current of 0.01 A/gel for 100 min, and stain with Coomassie brilliant blue. The concentration of purified proteins was determined using a BCA Protein Assay Kit (Solarbio Life Science, China).

### Enzyme assays

The enzyme activities were assessed using the artificial substrate Asp-*ρ*NA. A standard reaction was conducted in a 96-well plate on ice, with the addition of 120 μL of 50 mM imidazole (pH 7.5), 15 μL of 10 mM Asp-*ρ*NA (dissolved in DMSO), and 15 μL of a moderately diluted purified enzyme, resulting in a total volume of 150 μL per well. The contents were thoroughly mixed and then incubated at 40 ℃ for 10 min. The change in absorbance at 405 nm was measured before and after the incubation period. Each measurement was prepared in triplicate to ensure accuracy and reproducibility.

The optimal temperature for dipeptidase E activity was determined at different incubation temperatures from 25 to 55 °C based on the standard reaction. Subsequently, the optimal pH was assessed at the optimal temperature of 40 °C, using buffers with different pH values: pH 5.0 to 7.0 (100 mM citric acid−disodium hydrogen phosphate buffer), pH 6.0 to 7.5 (50 mM imidazole buffer), pH 7.5 to 9.0 (50 mM Tris-HCl buffer), and pH 9.0 to 10.0 (50 mM glycine-NaOH buffer).

To evaluate thermal stability, the purified enzyme was incubated at various temperatures (ranging from 10 to 80 °C) for 1 h. For pH stability assessment, the enzyme was incubated with different buffers at pH values ranging from 3.0 to 10.0 at 4 °C for 1 h. The residual activity was measured with the standard reaction. The effects of metal ions on Orf2 activities were investigated with buffers containing 1 mM of various metal ions (K^+^, Mg^2+^, Ni^2+^, Cu^2+^, Co^2+^, Ca^2+^, Mn^2+^, Zn^2+^, Fe^2+^, Fe^3+^). Similarly, the effects of organic solvents were examined with buffers containing 30% (v/v) of different organic solvents: acetic acid (AC), acetonitrile (ACN), dimethyl sulfoxide (DMSO), ethanol (EtOH), glycerol (Gl), isopropanol (IPA), and n-butanol (n-Bu). Furthermore, the effects of surfactants were assessed using buffers containing 1% or 5% (v/v) of different surfactants: Nonidet P-40 (NP40), sodium dodecyl sulfate (SDS), Triton X-100, and Tween 80. The enzyme’s sensitivity to the serine hydrolase inhibitor phenylmethanesulfonyl fluoride (PMSF) was tested by adding 1 mM or 5 mM PMSF to the reaction buffer. To determine the kinetic parameters of Orf2, the dipeptidase E activity was tested using various concentrations of Asp-*ρ*NA (ranging from 3.91 to 500 μM) in the standard reaction. *K*m, *V*max, *K*cat, and specific activities were calculated based on the Michaelis-Menten model using Excel.

### Substrate specificity analysis

The activity evaluation of dipeptidase E with Asp-X dipeptidases was performed using the improved cadmium-ninhydrin method (Doi et al. 1981). The Asp-X substrates used in the assay were Asp-Leu, Asp-His, Asp-Ala, Asp-Pro, Asp-Gly-Gly, and isoAsp-Leu (Leonbio, Nanjing, China). The reaction was carried out in 200 μL PCR tubes, where 120 μL of 50 mM imidazole (pH 7.5), 15 μL of 10 mM Asp-X (dissolved in 50 mM imidazole, pH 7.5), and 15 μL of 0.2 µM enzyme were added and thoroughly mixed. The mixture was then incubated at 40 ℃ for 10 min. After the incubation, the tubes were immediately placed on ice, and 50 μL of the reaction solution was transferred to 450 μL of 50 mM imidazole (pH 7.5) in 1.5 mL centrifuge tubes. To initiate color development, 1 mL of cadmium-ninhydrin solution was added to the tubes. The contents were vortexed and incubated at 84 °C for 5 min. After incubation, the tubes were cooled to room temperature on ice, and the absorbance was measured at 507 nm. The concentration of the product was then calculated using a standard curve.

### Bioinformatic analysis

The amino acid sequences of dipeptidases E, most of which have been experimentally verified, were obtained from the NCBI and UniProt databases. To determine their family memberships, investigations were carried out using the MEROPS database (Rawlings et al. 2018) available at https://www.ebi.ac.uk/merops/. The putative model of Orf2 was obtained from UniProt, which was predicted by AlphaFold2, and the ligand was Asp-*ρ*NA. Molecular docking was performed with AutoDock Vina (version 1.1.2) (Trott and Olson 2010) with the exhaustiveness 12. A search area with 25 × 25 × 25 Å^3^ was set in the studied protein binding site.

### Disruption of *orf2* in *Streptomyces* sp. 139

Using the *S* sp. 139 chromosome as a template, a 1000-bp upstream region of *orf2* (designated as F1) was amplified with primers F1-F/F1-R, while a 1000-bp downstream region of *orf2* (designated as F2) was amplified with primers F2-F/F2-R. The PCR protocol involved an initial denaturation at 98 ℃ for 3 min, followed by 30 cycles of 10 s at 98 ℃, 5 s at 60 ℃, and 60 s at 72 ℃. Finally, there was an extension step of 300 s at 72 ℃. A 1.1-kb fragment containing the thiostrepton resistance gene (*tsr*) was obtained by digesting the T-*tsr* plasmid with *Xba*I.

The three DNA fragments (F1, *tsr*, and F2) were ligated using the Gibson Assembly® Master Mix (NEB, England). This resulted in a 3-kb fragment, which was then inserted into the *Eco*RI-*Hin*dIII sites of the pKC1139 plasmid (Bierman et al. 1992) to create the *orf2*-disruption vector, named pKC1139-Δ*orf2*. After propagation in *E. coli* ET12567 (Liu et al. 2019), pKC1139-Δ*orf2* was introduced into *S* sp. 139 using polyethylene glycol (PEG)-mediated protoplast transformation (Geng et al. 2022). The plates were incubated at 28 ℃ for 20 h and then overlaid with soft R2YE (0.7% agar) containing 40 μg/L of kanamycin. The pKC1139-Δ*orf2* plasmid contains a temperature-sensitive *Streptomyces* replication origin that cannot replicate when the temperature reaches 34 ℃. The transformants were first incubated at 28 ℃ for 3 to 5 days until pinpoint-sized colonies appeared and were then shifted to 37 ℃ for further incubation on 139 media. The mutants resulting from double-crossover homologous recombination grew out of the original pinpoint-sized colonies within 2 to 4 days. The disruption of *orf2* on the chromosome was confirmed by PCR and Southern blot (Geng et al. 2022), and the *orf2* disruption mutant was designated as strain *S* sp. 139Δ*orf2*.

### Analysis of crude polysaccharides

*S* sp. 139 fermentation and crude polysaccharide extraction were performed as previously described (Geng et al. 2022). A weighed amount of 15 mg of crude polysaccharides was taken, and ddH_2_O was added to create a 2 mg/mL solution. Then, 1/5 volume of Sevag reagent (chloroform to n-butanol = 4:1, v/v) was added. After vigorous shaking, the mixture was centrifuged at 4000 × *g* for 7 min following a 30-min interval. The precipitate was discarded, and the supernatant was collected. This process was repeated until no denatured protein was observed. The resulting solution was lyophilized, and a 1 mg/mL solution was prepared. The solution was then filtered through a 0.22 μm filter membrane and subjected to high-performance gel permeation chromatography (HPGPC) analysis. For HPGPC analysis, two Agilent GPC columns (PL aquagel-OH MIXED-H, 8 μm, 7.5×300 mm) were connected in series and eluted with ddH_2_O. The flow rate was set at 0.8 mL/min, and the column temperature was maintained at 30 ℃. An injection volume of 20 μL was used. The evaporation photodetector settings were as follows: evaporation temperature of 60 ℃, atomization temperature of 40 ℃, and a gas flow rate of 1.6 L/min.

### RNA-seq

*S* sp. 139 and the *orf2* mutant strain *S* sp. 139Δ*orf2* were separately cultured in 25 mL TSB medium at 28 ℃ for 36 h, with each culture performed in triplicate. After this initial culture, the cells were inoculated at a 1:20 (v/v) ratio into 50 mL of fresh TSB medium and incubated at 28 ℃ for an additional 24 h. Subsequently, the cells were harvested by centrifugation at 4000 × *g* for 10 min. The cell pellet was collected, rapidly frozen in liquid nitrogen, and then sent for RNA extraction and RNA sequencing services provided by Novogene (Beijing, China).

The RNA sequencing data obtained from the analysis have been deposited in the NCBI Sequence Read Archive (SRA) under the BioProject accession number PRJNA1010516 and can be accessed with the following link: https://www.ncbi.nlm.nih.gov/bioproject/PRJNA1010516.

## Results

### Sequence BLAST and structure prediction of Orf2 as a dipeptidase E

The *orf2 *gene is located within the Ebosin biosynthetic gene cluster in *S.* sp. 139 (Fig. 1a). The sequence alignment analysis reveals the potential that the Orf2 protein (GenBank: QER86497.1) may be a dipeptidase E (PepE, EC: 3.4.13.21), which is annotated as a member of the S51 protein family of alpha-aspartyl dipeptidases (MEROPS ID: S51.001). Proteins belonging to the dipeptidase E family exhibit the capability of hydrolyzing dipeptides containing N-terminal Asp residues, denoted as Asp-X (where X represents any amino acid). These proteins are classified as non-classical serine hydrolases and exhibit the typical catalytic triad “Ser-His-Glu/Asp” that is characteristic of serine hydrolases. Multiple sequence alignment of reported dipeptidase E family proteins was performed using ClustalW in MEGA 11 (Fig. 1b). Furthermore, the secondary structure analysis refers to the predicted structure of the Orf2 protein by AlphaFold2 (Fig. 1b) which reveals that each protein in the dipeptidase E family has more conservative alpha-helices and beta-sheets. Additionally, certain regions between specific secondary structures such as the GG sequence between β6 and α4, as well as the GXSAG sequence between β7 and α6, are highly conserved. The S51 homolog NEV86160 is present in the whole genome shotgun of *S*. *tendae* strain VITAKN (GenBank: JAAIFS01), but there is no homolog of Orf2, as well as there is no NEV86160 homolog that has not been found in* S*. sp. 139. More studies have found that some *Streptomyces* have two S51 family homologs (similar to Orf2 and NEV86160, respectively), while some have only one of the two, which reveals to some extent the diversity of the S51 family in *Streptomyces*.

The structure of the Orf2 protein predicted by AlphaFold2 was downloaded from UniProt and visualized by PyMOL (Fig. 1c). The structure analysis reveals that Orf2 consists of seven alpha-helices, which are interconnected by several beta-sheets and random coils, giving the protein its overall 3D structure. Ser134, His170, and Asp198 are positioned within the substrate pocket of the Orf2, suggesting their crucial role in catalysis. Specifically, His170 forms hydrogen bond interactions with both Ser134 and Asp198. The distance between His170 and Ser134 is measured to be 2.8 Å, while the distance between His170 and Asp198 is 2.7 Å.

Molecular docking of Orf2 and Asp-*ρ*NA was performed using AutoDock Vina (Fig. 1d). The docking results revealed that the Asp residue of Asp-*ρ*NA was inserted into a recess within the enzyme, and both S134 and H170 were found to be in close spatial proximity to the substrate. Additionally, a hydrogen bond interaction was observed between D147, which is near the substrate pocket and marked in pink, and the substrate. This interaction may play a role in the formation of the enzyme-substrate complex.

### Cloning, expression, and purification of Orf2 and mutant proteins in *E. coli*

The *orf2* gene (744 bp) is present in the *S* sp. 139 genome, and its location is specified as GenBank accession number CP043959.1, spanning from position 2537865 to 2538608 on the genome. Following its cloning, the recombinant Orf2 (GenBank accession number QER86497.1) was successfully expressed in *Escherichia coli* (*E. coli*) and purified (Fig. S1). The molecular weight of Orf2 was 31.87 kDa.

### Enzyme characterization of Orf2 as a dipeptidase E

**Fig. 2:**
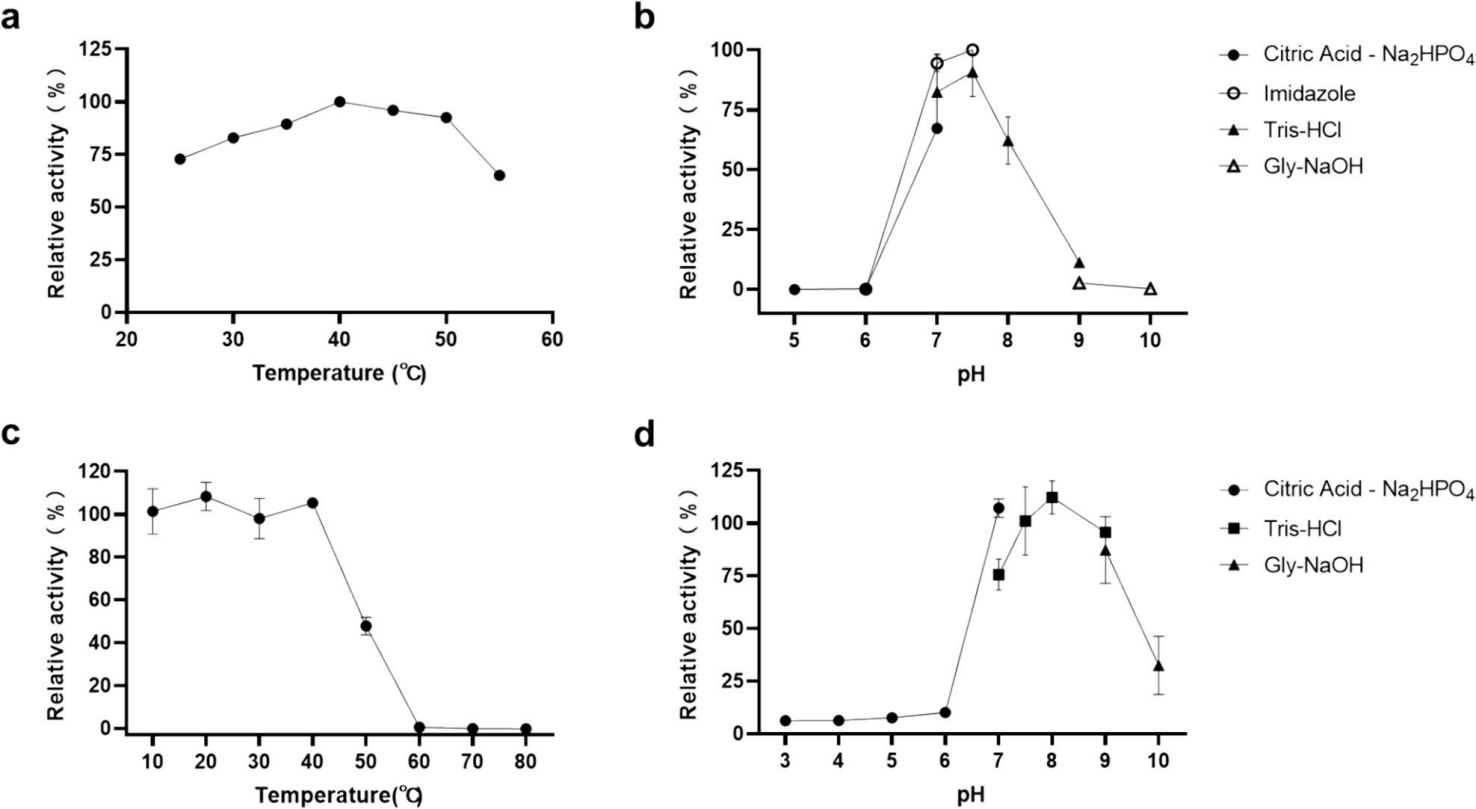
Optimum temperature (**a**), optimum pH (**b**), temperature stability (**c**), and pH stability (**d**) of Orf2

Through sequence alignment and structural analysis, it has been speculated that Orf2 from *S* sp. 139 may be a novel dipeptidase E. Dipeptidase E shows specificity toward dipeptides with an N-terminal Asp (Asp-X), and the enzyme activity can be determined by chromogenic substrate Asp-*ρ*NA (Lassy and Miller 2000; Yadav et al. 2019). The following enzyme activity assay results demonstrate that Orf2 can hydrolyze the chromogenic substrate Asp-*ρ*NA and its optimal enzymatic activity at a temperature of 40 °C. Moreover, Orf2 retains over 70% of its maximum activity within a broad temperature range of 25–50 °C (Fig. 2a). The purified enzyme exhibits its highest activity at pH 7.5 when tested in 50 mM imidazole buffer (Fig. 2b). Additionally, Orf2 retains considerable activity in the pH range of 7 to 9 when tested with different buffers. The thermal stability analysis of Orf2 reveals that it exhibits remarkable stability when incubated at temperatures ranging from 10 to 40 °C for 1 h. Even at 50 °C, about 45% of the enzyme’s activity remains after standing for 1 h (Fig. 2c). Concerning pH stability, Orf2’s activity is relatively unaffected after standing for 1 h in pH 7 to 9 solutions. However, the enzyme’s activity decreases significantly in solutions with more acidic or alkaline pH levels (Fig. 2d).

**Fig. 3:**
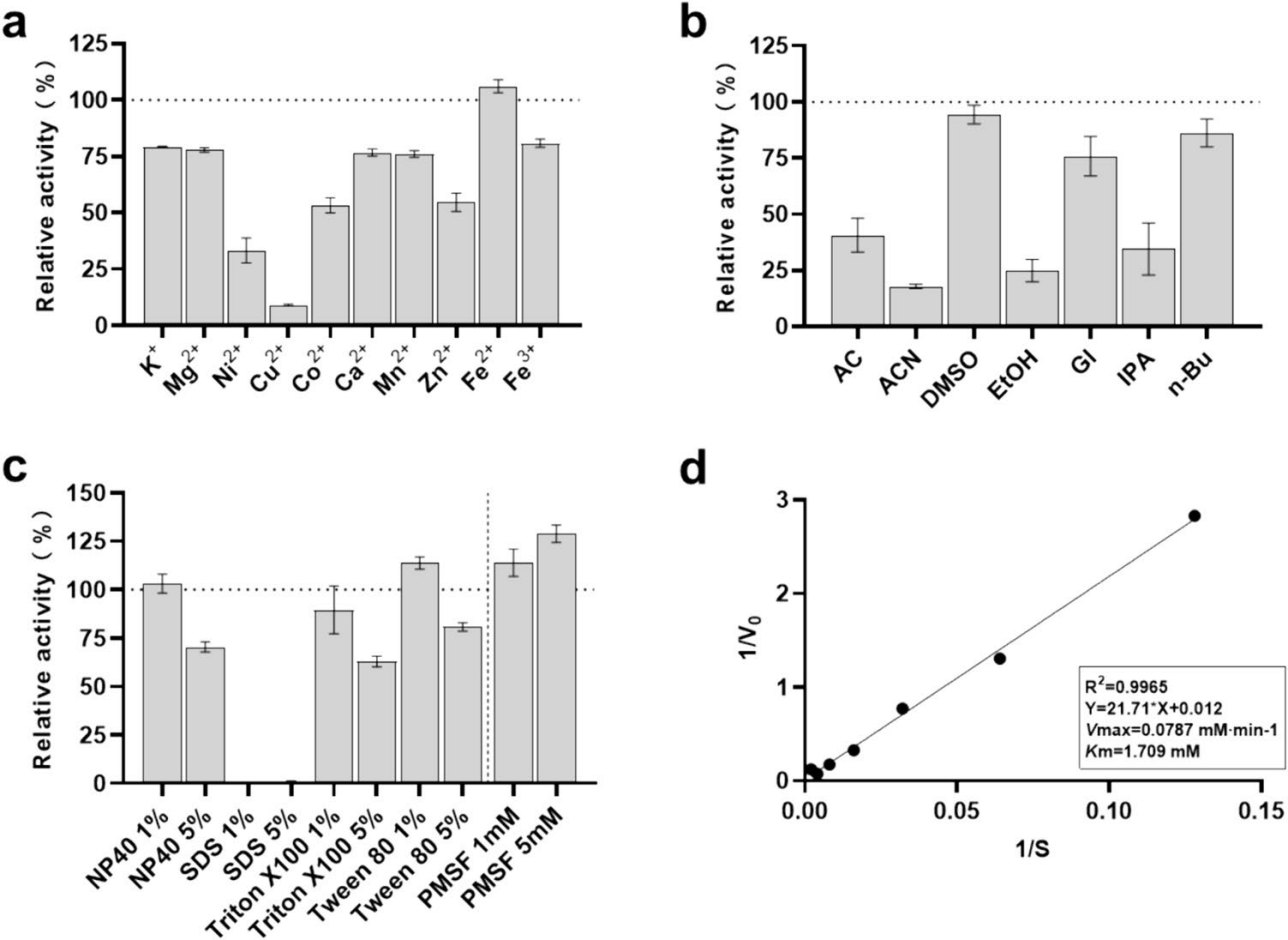
Effects of metal ions (**a**), organic solvents (**b**), surfactants, PMSF (**c**) on Orf2 and kinetic parameters of enzyme catalysis of Orf2 (**d**).

The effects of various metal ions and chemicals on Orf2 were examined (Fig. 3a). In the presence of Fe^2+^, Orf2 showed a slight increase in activity. However, the other metal ions tested did not have a significant promoting effect on its activity. On the contrary, K^+^, Mg^2+^, Ca^2+^, Mn^2+^, and Fe^3+^ slightly inhibited the enzyme activity, while Ni^2+^, Co^2+^, and Zn^2+^ exhibited stronger inhibitory activities. The presence of Cu^2+^ strongly inhibited Orf2’s activity. The influence of different organic solvents at a 30% final concentration was also evaluated. The enzyme activity was inhibited to varying degrees in the presence of these solvents. Notably, about 75% of the enzyme activity remained in the presence of 30% DMSO, glycerol, and n-butanol, while the enzyme retained approximately 25% activity in the presence of acetic acid, acetonitrile, ethanol, and isopropanol (Fig. 3b). When subjected to different surfactants, 1% NP40 and Triton X-100 did not significantly inhibit the enzyme’s activity, and even 1% Tween 80 slightly promoted its activity. However, 5% of NP40, Triton X-100, and Tween 80 exerted a certain inhibitory effect on the enzyme activity, though Orf2 still retained over 50% of its activity (Fig. 3c). The* V*max and *K*m of Orf2 are 0.0787 mM·min^−1^ and 1.709 mM, respectively (Fig. 3d). The *K*cat is 5.628 s^−1^, *K*cat/*K*m is 3.293 × 10^−3^ M^−1^·s^−1^, and specific activity of Orf2 is 1.690 ± 0.028 U/mg, respectively.

### Substrate specificity

In order to investigate the substrate specificity of Orf2, various peptides with Asp as the N-terminal residue (Asp-X) were employed as substrates, including Asp-Leu, Asp-His, Asp-Ala, Asp-Pro, Asp-Gly-Gly, and isoAsp-Leu (Table 2). The results demonstrated that Orf2 displayed the highest degradation activity toward Asp-Gly-Gly, followed by Asp-Leu, Asp-His, and isoAsp-Leu. However, the enzyme exhibited minimal or negligible activity on Asp-Pro and Asp-Ala. The findings suggest that dipeptidase E can degrade most dipeptides; however, its activity may be influenced by factors such as the size, hydrophobicity, and electrostatic interactions of the C-terminal amino acid residues in the substrate. Notably, Asp-Gly-Gly, being the smallest tripeptide, can effectively fit into the substrate pocket of certain dipeptidase E family proteins, making it a substrate that can be readily degraded by the enzyme.

**Table 2:** Substrate specificity of Orf2

Substrate	Relative activity (%) mean ± SEM
Asp-Gly-Gly	100.0 ± 2.343*
Asp-Leu	36.26 ± 1.341
Asp-His	15.05 ± 0.678
isoAsp-Leu	14.03 ± 2.145
Asp-Pro	0.669 ± 0.434
Asp-Ala	0.105 ± 0.006

^*^The specific activity of reducing Asp-Gly-Gly is 0.0995 ± 0.00330 U/mg

### Enzyme activity of Orf2 mutant proteins

**Fig. 4:**
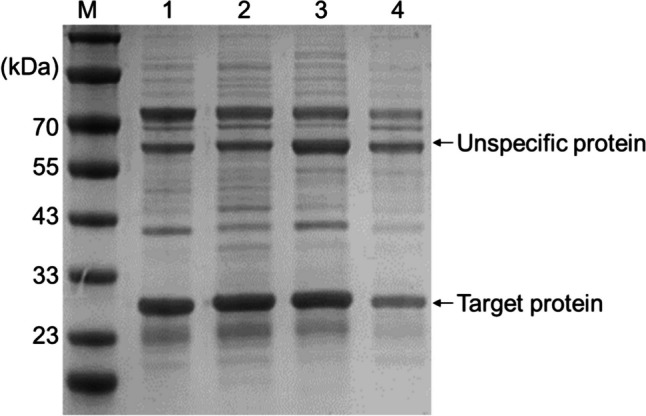
SDS-PAGE analysis of Orf2 mutant proteins. SDS-PAGE analysis of the Orf2 mutant proteins. M, Blue Plus® V Protein Marker. Lane 1, Orf2S134A. Lane 2, Orf2D147A. Lane3, Orf2H170A. Lane 4, Orf2D198A.

To further investigate the significance of the catalytic triad (S134, H170, and D198) and the D147 amino acid residue on enzyme activity, mutant proteins were generated and their enzymatic activities were analyzed. The four Orf2 mutant proteins, namely, Orf2S134A, Orf2H170A, Orf2D198A, and Orf2D147A, were purified (Fig. 4). The following enzymatic activity assay showed that all of these mutant proteins lost their activity (data not shown), indicating the essential residues in the enzymatic function of Orf2. Among the mutants, S134, H170, and D198 were identified as the putative “catalytic triad” of Orf2, responsible for its enzymatic activity. Moreover, D147, which was previously found through molecular docking to be in close proximity to the substrate pocket, was confirmed to be crucial for the enzyme’s activity. D147 likely affects the binding of the substrate to the enzyme, leading to the inactivation of the enzyme when mutated.

### *orf2* mutant strain construction and validation

To investigate the role of Orf2 in *S* sp. 139, the *S* sp. 139Δ*orf2* was created using a double crossover gene-replacement strategy. The plasmid pKC1139-Δ*orf2* was introduced into *S* sp. 139 through PEG-mediated protoplast transformation. After the transformation, numerous colonies (Thio^r^ Apr^s^) were randomly selected and confirmed through colony PCR. When the *orf2* gene was successfully replaced with the *tsr* fragment in the mutant strain, PCR amplification yielded fragments of 1751 bp (T1-F/R), 1578 bp (T2-F/R), and 1063 bp (T3-F/R). In contrast, the wild-type (WT) strain showed no amplification (N.D.) for these specific PCR fragments and, instead, yielded a fragment of 1400 bp (Fig. S2 a). The results of the PCR analysis confirmed the successful generation of the *orf2* mutant strain, as indicated by the absence of the expected PCR fragments corresponding to the *orf2* gene (Fig. S2 b). As shown in Fig. S2 a and c, a distinctive hybridization band of 2.9 kb was detected in the mutant strain and no band appeared in the wild type with the *tsr* as a probe. Southern blot confirmed that the *tsr* resistance cassette had been integrated into *orf2* in the strains.

### Analysis of crude polysaccharides

As shown in Table 3, compared with the *orf2* mutant strain (*S* sp. 139Δ*orf2*) and the WT, the yield of crude polysaccharide did not change significantly. The crude polysaccharides from both *orf2* mutant strain and the WT underwent HPGPC analysis. This analysis provided molecular weight distribution profiles for the respective polysaccharides (Fig. S3). The findings revealed a broader molecular weight distribution range for the crude polysaccharide isolated from *orf2* mutant strain in comparison to the WT. Notably, this broader range encompassed certain polysaccharide constituents with relatively lower molecular weights. These observations imply that the molecular weight distribution and structure of Ebosin derivatives underwent alterations subsequent to *orf2* mutant.

**Table 3:** Production of crude polysaccharide

Strain	Crude polysaccharide yield (g/L)
WT	1.761 ± 0.093
*S* sp. 139Δ*orf2*	1.738 ± 0.130

### Global transcriptional changes in *orf2* mutant strain

**Fig. 5:**
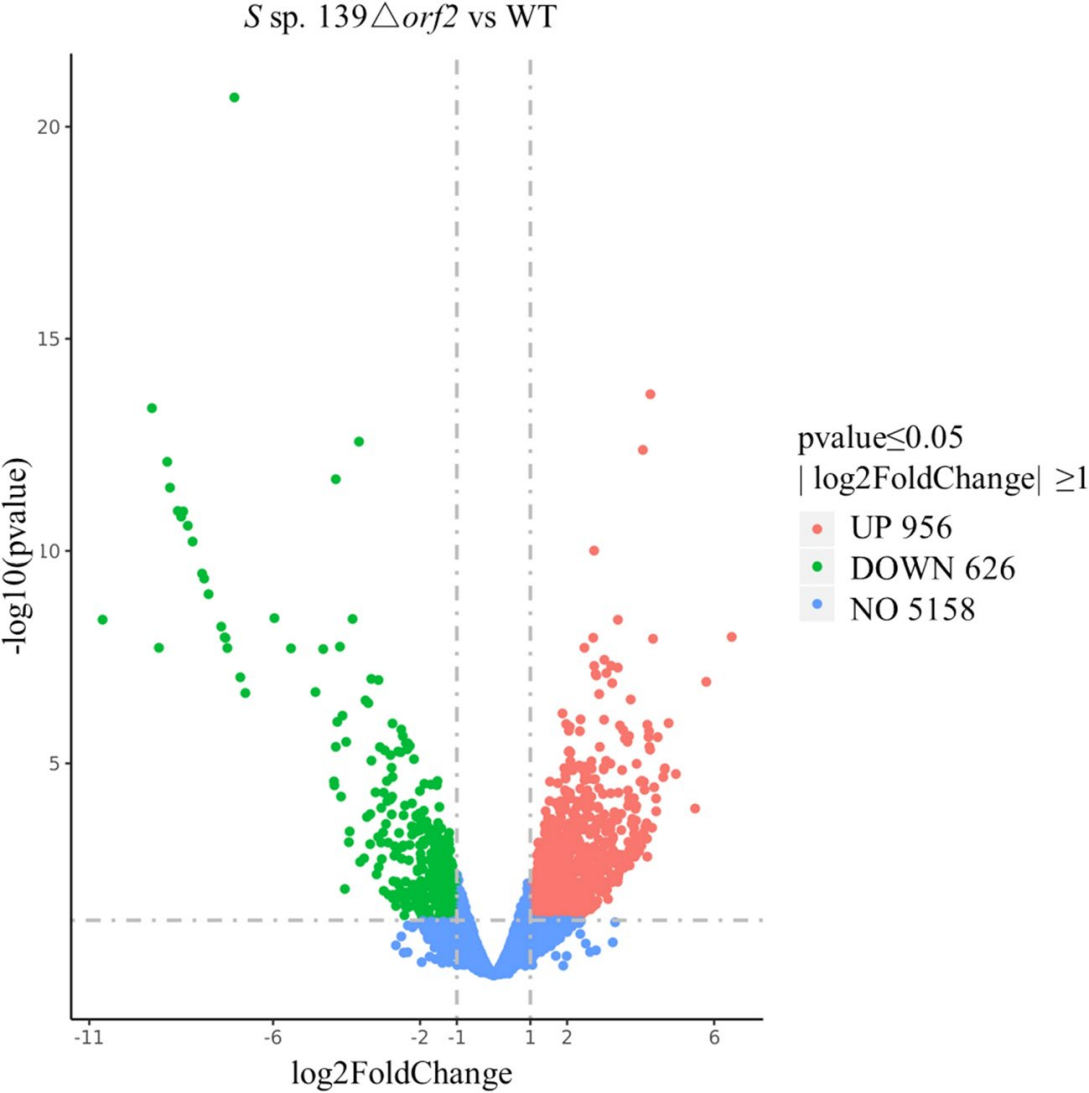
Volcano map of differentially expressed genes in *Streptomyces* sp. 139Δ*orf2*. The vertical dashed line is ∣log2FoldChange=1∣, and the horizontal dashed line is *p* value=0.05. Red represents transcription upregulated genes, green represents transcription downregulated genes, and blue represents genes with insignificant transcription changes.

Transcriptome analysis was conducted in triplicate for both the WT strain and the *orf2* mutant strain. By applying a cut-off of 2.0-fold difference in expression levels between the two strains, a total of 1582 genes were identified as differentially expressed. Among these genes, 956 were upregulated by more than 2.0-fold, while 626 were downregulated by more than 2.0-fold in the *orf2* mutant when compared with the WT strain (Fig. 5). These changes in gene expression were found to be statistically significant (*p* value ≤ 0.05). In contrast, 5158 genes showed no statistically significant differences in expression between the two strains. To gain further insights into the functions and pathways affected by the differential gene expression, gene-annotation enrichment and functional annotation clustering analyses of the differentially expressed genes (DEGs) were performed based on the NCBI genome annotation of *S* sp. 139 (Wang et al. 2010).

**Fig. 6:**
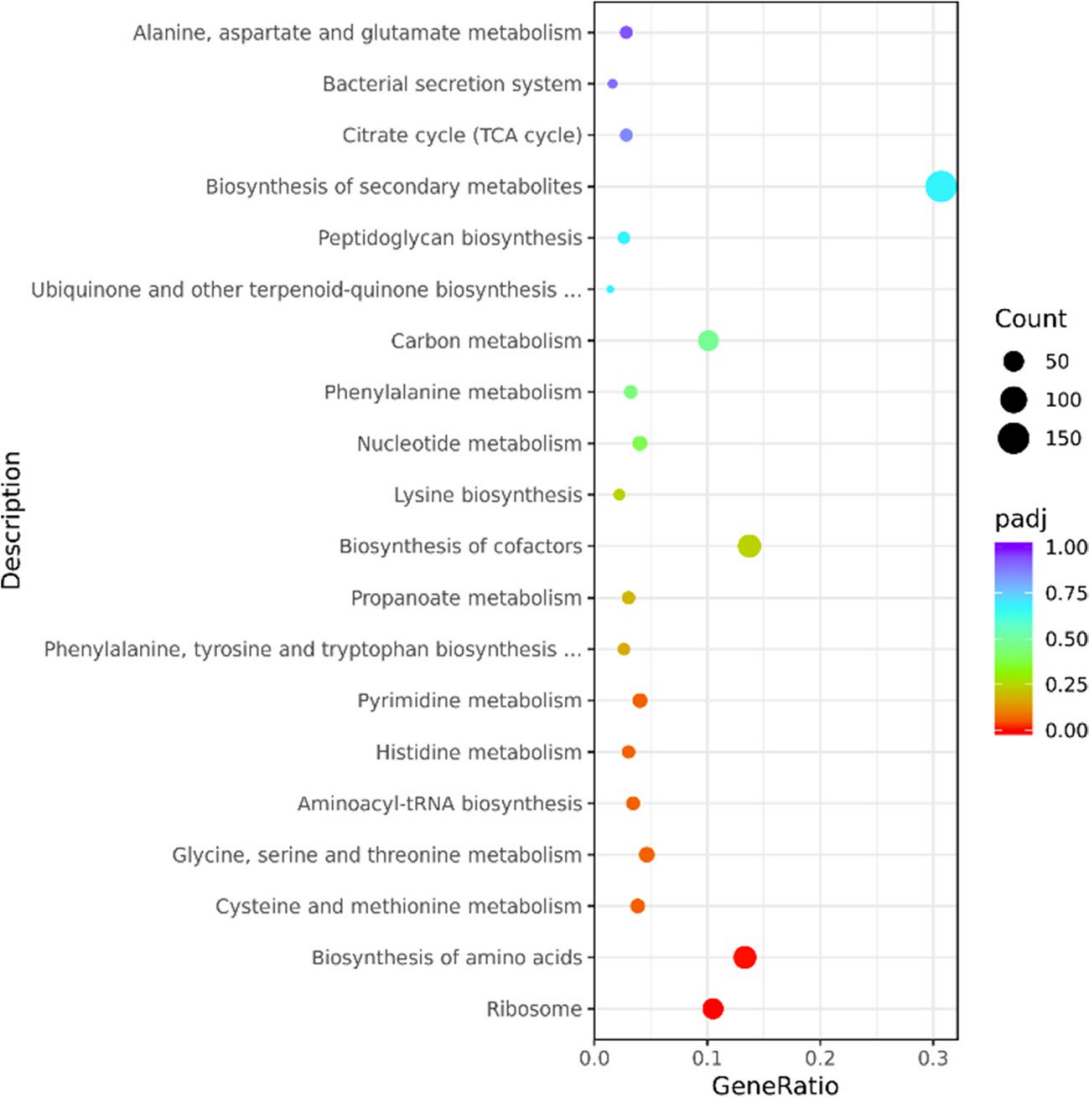
DEG number of the most enriched pathway.

The DEGs were found to be significantly enriched in 30 terms corresponding to Kyoto Encyclopedia of Genes and Genomes (KEGG) pathways (Fig. 6). These enriched pathways include ribosome, biosynthesis of amino acids, cysteine and methionine metabolism, glycine, serine, and threonine metabolism, aminoacyl-tRNA biosynthesis. According to the definitions of these KEGG pathways, it appears that many of the genes showing significant regulation are involved in processes related to ribosomal function, amino acid biosynthesis, and the metabolism of cysteine, methionine, glycine, serine, and threonine. The findings suggest that the *S* sp. 139Δ*orf2* strain exhibits increased activity in ribosome-related processes, aminoacyl tRNA biosynthesis, and certain amino acid metabolic pathways compared to the WT strain. It is plausible that the disruption of the *orf2* gene leads to be increased compensatory synthesis of peptides and proteins in *S* sp. 139.

### Analysis of differential genes combined with metabolic pathways

There is a single type of ribosome known as the 70S ribosome in *Streptomyces*. This ribosome is composed of two subunits, namely, the 50S large subunit and the 30S small subunit. The large subunit contains 5S RNA, 23S RNA, and 31 ribosomal proteins, while the small subunit is composed of 16S RNA and 21 ribosomal proteins (Zhu et al. 2019). In the context of the ribosomal pathway analysis, it was observed that the transcript levels of most ribosomal proteins were significantly upregulated in mutant strain (Fig. S4). This upregulation suggests that the mutant strain has an increased demand for protein synthesis.

Amino acids are crucial building blocks for protein synthesis in organisms, providing the necessary materials for bacterial growth, reproduction, and metabolic maintenance. In Fig. S5, the amino acid biosynthesis pathways of *S* sp. 139Δ*orf2* were found to be highly active for most amino acids, including histidine, valine, leucine, isoleucine, serine, cysteine, methionine, alanine, aspartic acid, glycine, asparagine, glutamic acid, arginine, proline, tryptophan, and threonine. These pathways play a significant role in the active synthesis of amino acids in the mutant strain. Among the amino acid synthesis pathways, lysine, tyrosine, and phenylalanine synthesis showed no significant changes. However, the expression of genes involved in the precursor biosynthesis of tyrosine (HPP) and phenylalanine increased, indicating that there might be an active synthesis of tyrosine and phenylalanine to some extent. Only lysine synthesis did not exhibit significant changes in the mutant strain. The active amino acid synthesis in *S* sp. 139Δ*orf2* may be a compensatory response to the loss of peptidase function caused by *orf2* disruption. By increasing amino acid synthesis, the mutant strain ensures an adequate supply of raw materials for peptide and protein synthesis despite the *orf2* disruption.

In the aminoacyl tRNA biosynthesis pathway, the expression levels of most tRNA synthetase genes in *orf2* mutant strain were found to be upregulated (Fig. S6). This upregulation suggests that the synthesis of aminoacyl tRNA, a crucial step in protein translation, is relatively active in the mutant strain. It indicates that the translation process in the mutant strain is highly active, and the strain is capable of rapidly synthesizing a large number of polypeptides and proteins. The increased expression of tRNA synthetase genes is essential for ensuring that the strain has an adequate supply of charged tRNA molecules, which are essential for the accurate and efficient translation of mRNA into proteins. By upregulating the expression of tRNA synthetases, the *orf2* mutant strain is equipped to meet the demands of its heightened translation activity and sustain its protein synthesis machinery.

The analysis of DEGs revealed that the expression levels of certain peptidase genes in the strains were significantly upregulated, while others showed no significant changes. Table S1 provides a list of 9 peptidase-encoding genes whose expression levels were significantly upregulated (log2FoldChange≥2). These genes belong to different peptidase families, including serine peptidase (4), metallopeptidase (4), and cysteine peptidase (1) families. Among them, F3L20_RS27855, which encodes an M6 family peptidase, showed the most significant upregulation. This particular peptidase has been reported to be associated with the sporulation process of *Bacillus cereus* (Charlton et al. 1999).

The upregulated peptidases in Table S1 exhibit diverse substrate specificities and functions: S37 family aminopeptidases are active on tripeptides or tetrapeptides. S8 family peptidases are endopeptidases. C40 family peptidases are dipeptidases. S11 family peptidases can degrade Ala-Ala. M1 family peptidases mainly act on the N-terminus of polypeptides and include many aminopeptidases. M20 family peptidases can degrade dipeptides or tripeptides. Most of these upregulated peptidase-encoding genes can degrade other peptides, suggesting a potential compensatory response to the *orf2* blockade. Notably, the transcriptome sequencing results did not identify any peptidase genes with significantly downregulated expression levels (log2FoldChange≤−2) except for *orf2* itself.

The analysis of DEGs revealed significant upregulation in the expression levels of three genes encoding the four amino acid ABC transporter permease in *S* sp. 139 (Table S2). This protein plays a role in mediating the transmembrane transport of amino acids. Additionally, the expression levels of genes encoding oligopeptide transport permease were also significantly upregulated. This type of protein is involved in the transmembrane transport of oligopeptides, including dipeptides. Moreover, the amino acid ABC transporter permease encoded by gene F3L20_RS32195 showed downregulation in its expression level. The observed upregulation in the expression levels of these transporter proteins suggests that the strain is more active in the transport processes of oligopeptides, dipeptides, and amino acids. The increased expression of these transporters may be a compensatory response to the disruption of *orf2* and its role in enzymatic hydrolysis, indicating that the strain is enhancing its capacity to import and utilize external sources of peptides and amino acids to maintain its metabolism and growth. Further research is needed to investigate whether the downregulated F3L20_RS32195 is a specific transporter for the enzymatic hydrolysis product of Orf2. Understanding the specific transport mechanisms and substrates of these transporter proteins can provide valuable insights into the overall metabolic and regulatory changes that occur in *orf2* mutant strain in response to *orf2* disruption.

As shown in Table S3, the expression of genes encoding seven F0F1 ATP synthase subunits in *S* sp. 139 genome annotations was significantly upregulated to varying degrees. This upregulation suggests that the ATP synthesis process in *orf2* mutant strain is more active. The increased expression of these ATP synthase subunits indicates that the strain has a higher demand for energy, and as a result, a large amount of ATP needs to be synthesized to meet this increased demand. ATP serves as the primary energy currency in cells, providing energy for various cellular processes such as biosynthesis, active transport, and cell division. The upregulation of ATP synthase subunits indicates that *orf2* mutant strain is undergoing significant metabolic changes to compensate for the disruption of *orf2* and to maintain its energy homeostasis. This heightened ATP synthesis activity reflects the strain’s efforts to support its increased metabolic demands and growth in response to the loss of *orf2* function. In summary, the upregulation of genes encoding F0F1 ATP synthase subunits in *orf2* mutant strain indicates a higher demand for energy in the blocked strain, and it highlights the strain’s active response to maintain its metabolic processes and growth in the absence of *orf2*.

## Discussion

Orf2 has been characterized as an aspartyl dipeptidase E in vitro, which can degrade the chromogenic substrate Asp-*ρ*NA, several dipeptides, and one tripeptide substrate. Slightly different with aspartyl dipeptidase E (PepE) from *Salmonella typhimurium* and *Xenopus laevis* (Lassy and Miller 2000), Orf2 displays a higher relative activity with Asp-Gly-Gly and isoAsp-Leu substrates and only moderate-level activity with Asp-Leu and Asp-His. The active site of Orf2 contains a Ser-His-Asp catalytic triad, which is incompletely similar to eukaryotic PepE from *Xenopus laevis* and prokaryotic PepE from *Salmonella typhimurium* with a Ser-His-Glu catalytic triad (Kumar et al. 2022; Lassy and Miller 2000). In addition, the Asp147 residue of Orf2 is also proven to be critical for the enzyme’s activity through point mutation. To the best of our knowledge, it is the first report that the dipeptidase E from *Streptomyces* has been characterized. Notably, the Orf2 exhibits tolerance for some organic solvents and surfactants, implying its potential utility as an industrial enzyme.

It had been reported that dipeptidase mutants in *Salmonella typhimurium*, *Metarhizium acridum*, and *Pseudomonas aeruginosa* were tested for growth on dipeptides as amino acid sources (Carter and Miller 1984). In our research, in vivo transcriptome analysis revealed the upregulation of genes associated with ribosomes, amino acid biosynthesis, and aminoacyl-tRNA biosynthesis in the *orf2* mutant. The upregulation of genes encoding oligopeptide, dipeptide, and amino acid transporters supports the idea of compensatory responses to the disruption of *orf2*, possibly to meet the demand for amino acid raw materials. Furthermore, 15 of the 16 SpoIIE family protein and all of Chaplin protein-coding genes are downregulated in the *orf2* mutant (data not shown), which were instrumental in the formation of aerial structures by the *Streptomyces* (Anderl et al. 2020; Ekkers et al. 2014; Zhang and Shi 2004). We speculate Orf2 may be involved in spore and mycelia formation in *S* sp. 139, but it still needs further research.

Compared with *orf2* mutant strain and WT, the yield of crude polysaccharide did not change significantly. The HPGPC analysis revealed that crude polysaccharides from the *orf2* mutant strain exhibited a wider range of molecular weight distribution compared to the WT strain. This suggests that Orf2 links nutrient stress to secondary metabolism by *S* sp. 139. As only one reports that exopolysaccharide yield was related to dipeptidase activities in *Lactobacillus* (Meng et al. 2018), the study has provided important insights into the role of Orf2 in *S* sp. 139 and helped for further research on the biological functions of peptidases in metabolism of peptide and polysaccharide.

## Supplementary Information

Below is the link to the electronic supplementary material.

Supplementary file1 (PDF 750 KB)

## Data Availability

All documents and additional data are available from the corresponding author upon reasonable request. RNA sequencing data have been submitted to the Sequence Read Archive Database of National Center for Biotechnology Information under the accession PRJNA1010516, and the data can be accessed using the following link: https://www.ncbi.nlm.nih.gov/bioproject/PRJNA1010516.
