# Supplementary material for: Characterization and functional evidence for Orf2 of Streptomyces sp. 139 as a novel dipeptidase E
Source: Appl Microbiol Biotechnol. 2024 May 8;108(1):326. doi: 10.1007/s00253-024-13161-y (PMC11078827; doi:10.1007/s00253-024-13161-y)
Supplement: Supplementary file 1 — Supplementary file1 (PDF 750 KB) [file 253_2024_13161_MOESM1_ESM.pdf]

Supplementary Information

Characterization and functional evidence for Orf2 of *Streptomyces* sp. 139 as a novel dipeptidase E.

Zhe Liu[1, 2], Kemeng Li[1, 2], Jialin Li[1, 2], Zhuochen Zhuang[1, 2], Lianhong Guo[1, 2], Liping Bai[1, 2*]

[1] CAMS Key Laboratory of Synthetic Biology for Drug Innovation, Institute of Medicinal Biotechnology, Chinese Academy of Medical Sciences & Peking Union Medical College, Beijing 100050, China

[2] NHC Key Laboratory of Biotechnology of Antibiotics, Institute of Medicinal Biotechnology, Chinese Academy of Medical Sciences & Peking Union Medical College, Beijing 100050, China

Corresponding author：Prof. Liping Bai

Phone: +861063013336; E-mail: lipingbai1973@163.com, bailiping@imb.pumc.edu.cn

Correspondence should be addressed to: Institute of Medicinal Biotechnology, Chinese Academy of Medical Sciences & Peking Union Medical College, 100050，1 Tian Tan Xi Li, Beijing 100050, China

Table S1 Expression levels of significantly upregulated peptidase coding genes (log2FoldChange≥2)

Gene_id	log2FoldChange	p-Value	Pfam	Gene_description
F3L20_RS27855	3.68577	6.53E-05	PF05547	M6 family peptidase
F3L20_RS27130	3.65849	1.00E-04	PF05576	S37 family peptidase
F3L20_RS04500	3.55891	1.46E-03	PF00082	S8 family peptidase
F3L20_RS24255	3.16802	2.47E-04	PF00877	C40 family peptidase
F3L20_RS00325	3.02962	7.40E-04	PF00768	S11 family peptidase
F3L20_RS24445	2.88784	2.37E-03	PF00883	M17 family peptidase
F3L20_RS26635	2.84759	1.98E-03	PF11838	M1 family peptidase
F3L20_RS08700	2.32071	1.12E-03	PF00082	S8 family peptidase
F3L20_RS22500	2.20113	3.58E-03	PF01546	M20 family peptidase

Table S2 The expression of oligopeptides, dipeptides and amino acid transferases coding genes

Gene_id	log2FoldChange	p-Value	Pfam	Gene_description
F3L20_RS05800	3.64513	3.10E-06	PF00528	amino acid ABC transporter permease
F3L20_RS07915	3.24702	5.23E-04	PF00528	amino acid ABC transporter permease
F3L20_RS07920	2.88639	2.11E-03	PF00528	amino acid ABC transporter permease
F3L20_RS32195	-1.35932	2.52E-02	PF00528	amino acid ABC transporter permease
F3L20_RS05800	3.64513	3.10E-06	PF00528	amino acid ABC transporter permease
F3L20_RS06610	2.69791	8.56E-03	PF12911	oligopeptide transport permease
F3L20_RS05045	2.22275	9.33E-03	PF12911	oligopeptide transport permease
F3L20_RS06630	3.81674	6.62E-05	PF08352	oligopeptide/dipeptide transporter
F3L20_RS05065	3.49627	1.44E-05	PF08352	oligopeptide/dipeptide transporter
F3L20_RS06625	3.47494	3.74E-04	PF08352	oligopeptide/dipeptide transporter
F3L20_RS05060	2.80960	2.20E-04	PF08352	oligopeptide/dipeptide transporter

Table S3 The expression of ATP synthase coding genes

Gene_id	log2FoldChange	p-Value	Pfam	Gene_description
F3L20_RS06140	3.03740	1.08E-02	PF00430	F0F1 ATP synthase subunit B
F3L20_RS06155	2.99991	8.84E-03	PF00231	F0F1 ATP synthase subunit gamma
F3L20_RS06160	2.78124	2.12E-02	PF00306	F0F1 ATP synthase subunit beta
F3L20_RS06150	2.76311	1.93E-02	PF02874	F0F1 ATP synthase subunit alpha
F3L20_RS06145	2.44886	2.65E-02	PF00213	F0F1 ATP synthase subunit delta
F3L20_RS06130	2.32692	2.30E-02	PF00119	F0F1 ATP synthase subunit A
F3L20_RS06165	2.07781	2.98E-02	PF02823	F0F1 ATP synthase subunit epsilon

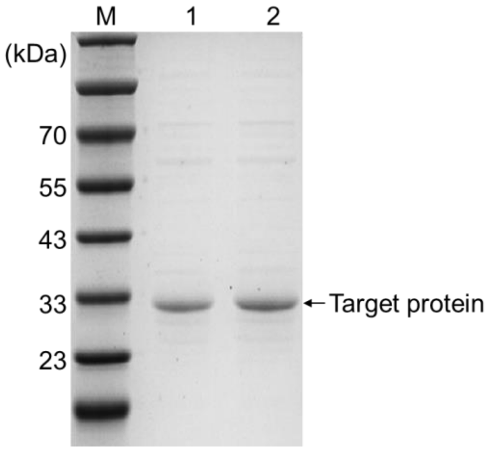

Fig.S1 SDS–PAGE analysis of the Orf2. M, Blue Plus® V Protein Marker. Lanes 1-2, Orf2.

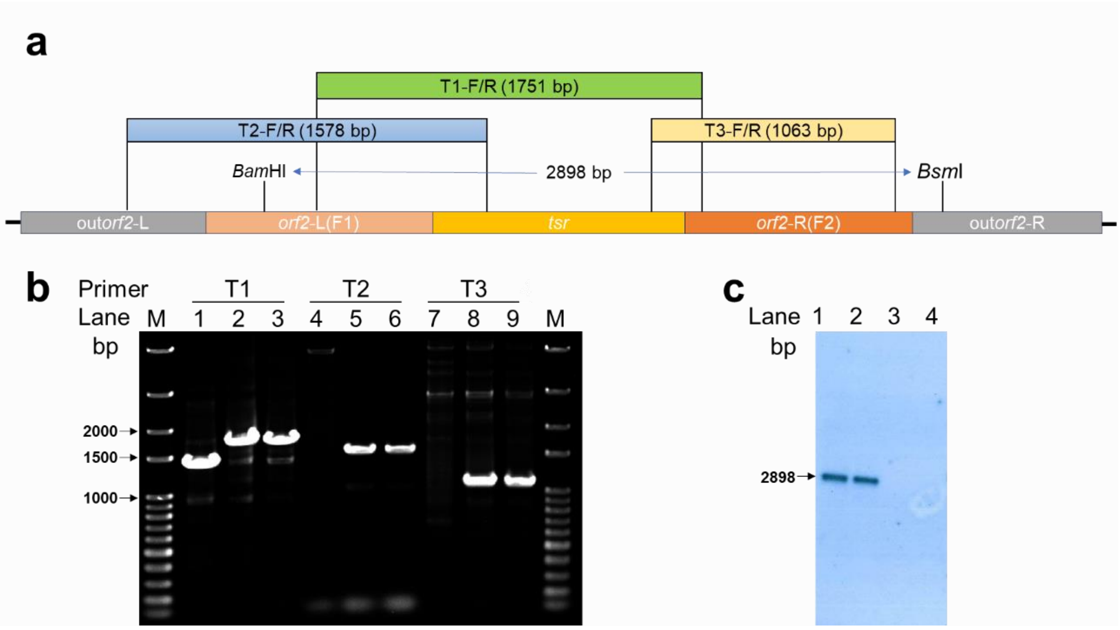

Fig. S2 *orf2* mutant strain construction and validation

Fig. S2 a: The primers and restriction enzyme cleavage sites used for verification on the mutant strain genome. b: Lane 1, 2 and 3 were used T1-(F/R) primers for PCR validation of WT, mutant 1 and mutant 2, respectively; Lane 4, 5 and 6 were used T2-(F/R) primers for PCR validation of WT, mutant 1 and mutant 2, respectively; Lane 7, 8 and 9 were used T3-(F/R) primers for PCR validation of WT, mutant 1 and mutant 2, respectively. c: Southern blot autoradiograph of *orf2* mutants and WT with *tsr* as a probe. Lane 1 and 2 were chromosome DNA of *orf2* mutants digested with *Bam*HI and *Bsm*I; Lane 3 and 4 were chromosome DNA of WT digested with *Bam*HI and *Bsm*I.

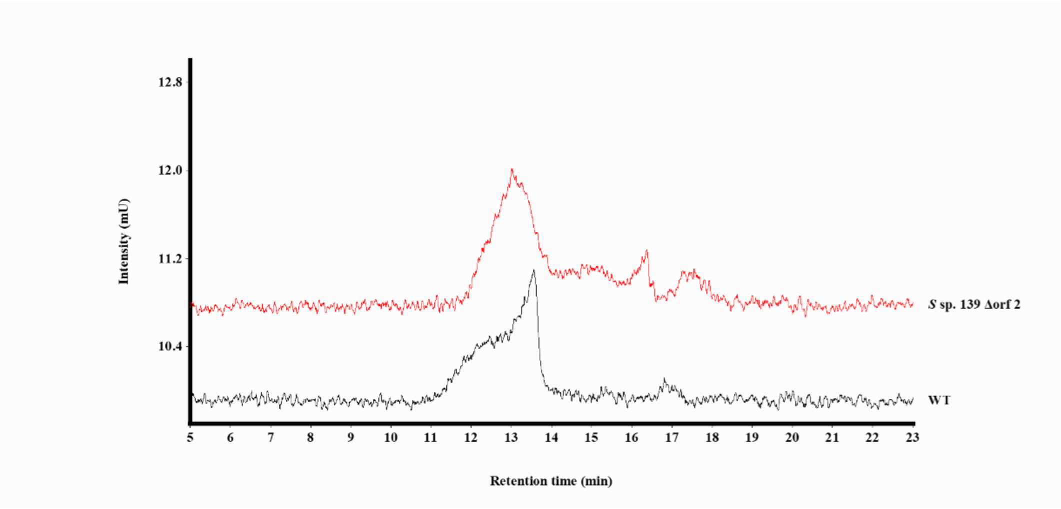

Fig. S3 Molecular distribution of crude polysaccharide from *Streptomyces* sp. 139△ *orf2* and WT strain via GPC analysis

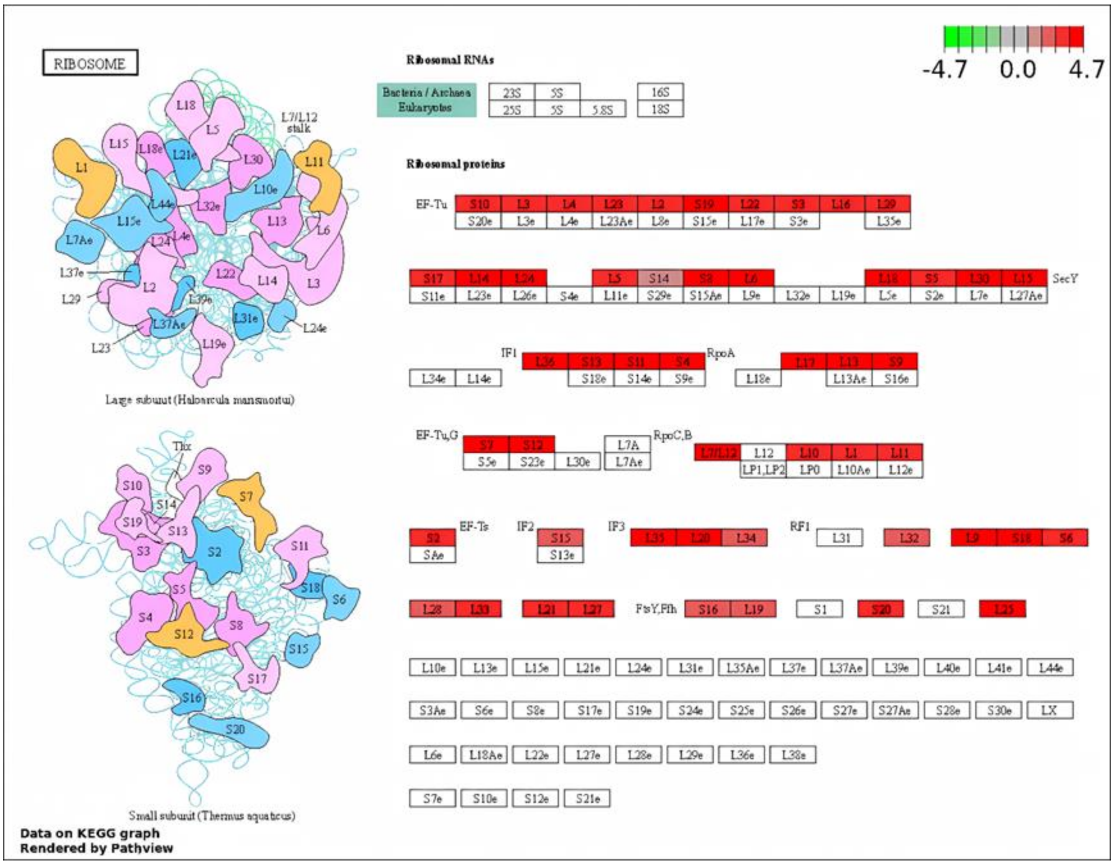

Fig. S4 Expression of key genes of ribosome pathway

The color of the square to which the gene belongs represents its expression level, which increases sequentially from green (log2foldchange=-4.7) to red (log2foldchange=4.7).

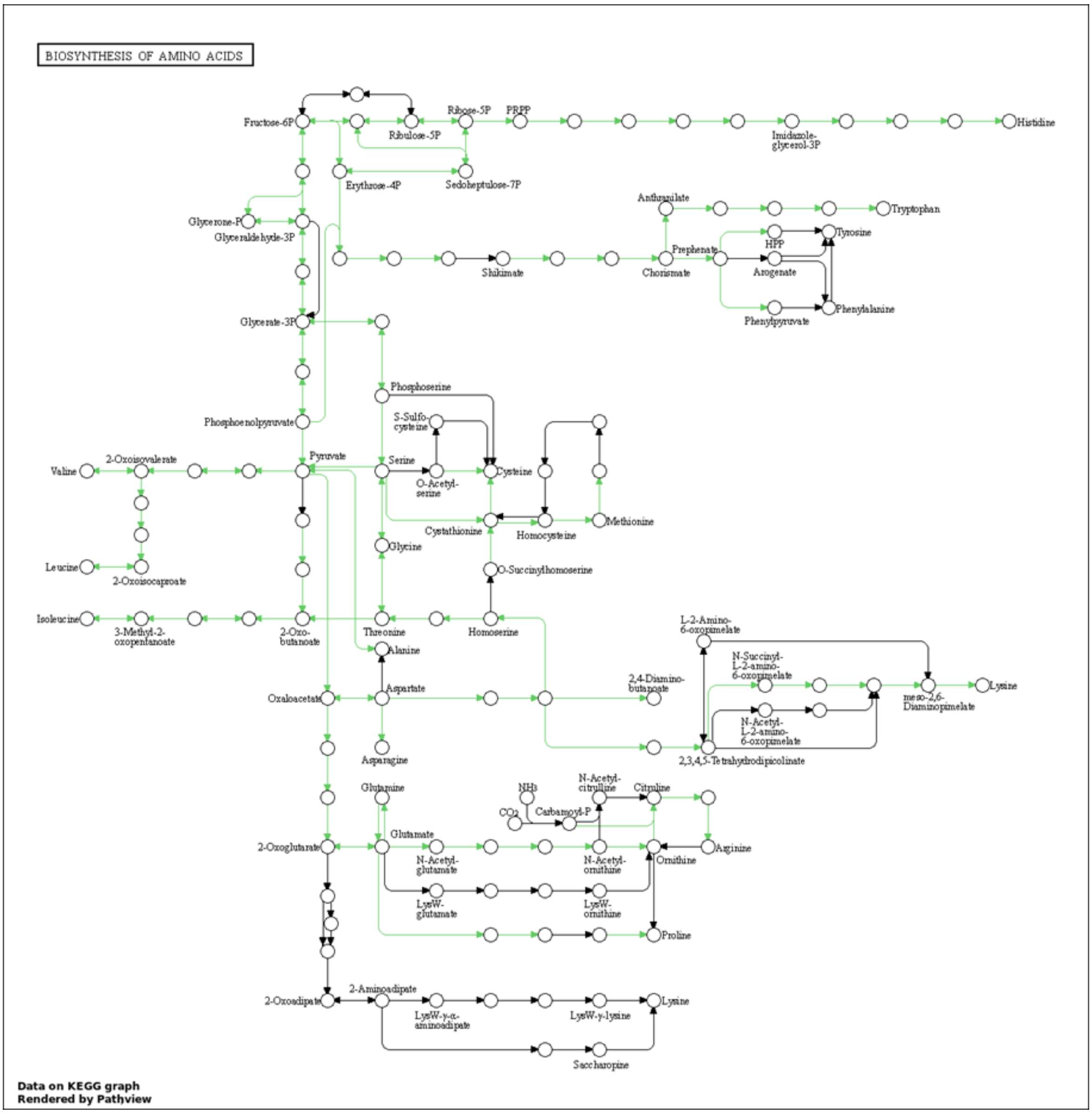

Fig. S5 Amino acid biosynthetic pathway

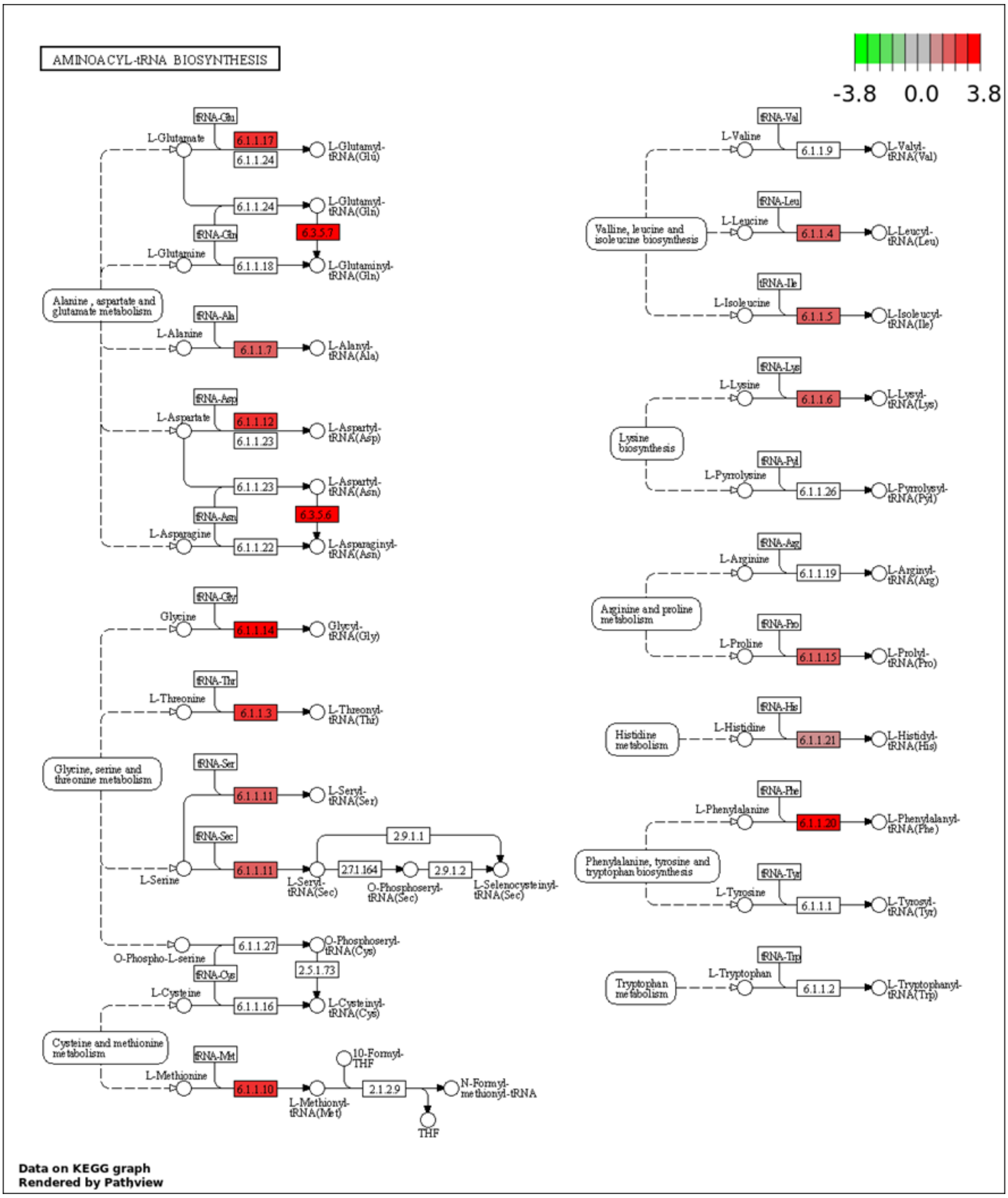

Fig. S6 Aminoacyl tRNA synthesis pathway
